# An optimized protocol for microarray validation by quantitative PCR using amplified amino allyl labeled RNA

**DOI:** 10.1186/1471-2164-11-542

**Published:** 2010-10-07

**Authors:** Céline Jeanty, Dan Longrois, Paul-Michel Mertes, Daniel R Wagner, Yvan Devaux

**Affiliations:** 1 Laboratory of Cardiovascular Research, Centre de Recherche Public-Santé, Luxembourg; 2 Department of Anesthesia and Intensive Care, Hopital Bichat-Claude-Bernard, Université Paris-VII, France; 3 Department of Anesthesia and Intensive Care, Centre Hospitalier et Universitaire de Nancy, Hôpital Central, Nancy, France; 4 Division of Cardiology, Centre Hospitalier, Luxembourg

## Abstract

**Background:**

Validation of microarrays data by quantitative real-time PCR (qPCR) is often limited by the low amount of available RNA. This raised the possibility to perform validation experiments on the amplified amino allyl labeled RNA (AA-aRNA) leftover from microarrays. To test this possibility, we used an ongoing study of our laboratory aiming at identifying new biomarkers of graft rejection by the transcriptomic analysis of blood cells from brain-dead organ donors.

**Results:**

qPCR for ACTB performed on AA-aRNA from 15 donors provided Cq values 8 cycles higher than when original RNA was used (P < 0.001), suggesting a strong inhibition of qPCR performed on AA-aRNA. When expression levels of 5 other genes were measured in AA-aRNA generated from a universal reference RNA, qPCR sensitivity and efficiency were decreased. This prevented the quantification of one low-abundant gene, which was readily quantified in un-amplified and un-labeled RNA. To overcome this limitation, we modified the reverse transcription (RT) protocol that generates cDNA from AA-aRNA as follows: addition of a denaturation step and 2-min incubation at room temperature to improve random primers annealing, a transcription initiation step to improve RT, and a final treatment with RNase H to degrade remaining RNA. Tested on universal reference AA-aRNA, these modifications provided a gain of 3.4 Cq (average from 5 genes, P < 0.001) and an increase of qPCR efficiency (from -1.96 to -2.88; P = 0.02). They also allowed for the detection of a low-abundant gene that was previously undetectable. Tested on AA-aRNA from 15 brain-dead organ donors, RT optimization provided a gain of 2.7 cycles (average from 7 genes, P = 0.004). Finally, qPCR results significantly correlated with microarrays.

**Conclusion:**

We present here an optimized RT protocol for validation of microarrays by qPCR from AA-aRNA. This is particularly valuable in experiments where limited amount of RNA is available.

## Background

Gene expression profiling using microarrays has rapidly become an analytical tool of choice for translational research laboratories. Genome-wide or more dedicated microarrays are generally used as a fishing expedition to identify candidate genes or pathways that can be used either for their prognostic performance and/or for their therapeutic potential in many diseases. The technique relies on the relative quantification of mRNA expression in cells or tissues. Circulating blood cells can be used as an alternative to tissue biopsies when these are not available. This alternative nevertheless assumes that a systemic biosignature of the pathological state exists and can be assessed through gene expression profiling of blood cells. Consistently, while biosignatures of blood cells were originally reported to be a useful prognostic tool for acute myeloid leukemia [1,2], several studies later showed that these biosignatures can also aid in the development of biomarkers of several diseases affecting vital organs such as the brain [3] and the coronary arteries [4]. Both peripheral blood mononuclear cells [3,4] and whole blood cells [5] have been used in such profiling experiments. One has nonetheless to keep in mind that the method of RNA collection, either from blood cells using the PAXgene™ technology for instance [6], or from buffy coats [3,4], is a critical variable when designing research protocols using microarray studies [5]. The PAXgene™ system is attractive because it stabilizes RNA immediately after collection without the need of rapidly isolating the leukocyte compartment. This is particularly relevant when designing clinical protocols in which patients are included any time of the day (patients with acute myocardial infarction for instance). In addition, this system requires only a very limited volume of blood. However, the reliability of this system to consistently detect all gene transcripts may be questioned [7].

In addition to the type of blood collection, every steps of the microarray technique can influence the quality of the results. When minute starting amounts of RNA are available, additional steps of amplification have to be performed [8,9]. This scenario is frequent when using the PAXgene™ system since RNA is extracted from only 2.5 mL of blood. Such RNA is generally processed through a multiple steps procedure to generate amplified amino allyl RNA (AA-aRNA) coupled with fluorescent dyes. First, RNA is reverse transcribed, then amplified with incorporation of amino allyl UTP (AA-UTP) to serve as an arm to facilitate dye binding, and finally coupled with fluorescent dyes before hybridization onto microarrays. This fastidious protocol introduces supplementary bias in the microarray technique, sometimes leading to false positive discovery and erroneous results [10-13]. Some alternatives have been developed, such as the Universal Linkage System technology (Kreatech Diagnostics, Amsterdam, The Netherlands) or the Ovation^® ^technology (NuGEN, San Carlos, CA, USA). Optimization of the amplification procedure has been tackled by previous investigators, such as Waddell et al. who reported two different methods for amplification of bacterial RNA to be assessed in microarray experiments [14]. A popular approach to balance the problem of false discovery is to validate microarray data using an independent technique, such as Northern blot hybridization, RNase protection assay or real-time quantitative PCR (qPCR), the latter being the more widely used [15]. Most commonly performed on cDNA obtained from reverse transcription (RT) of total RNA, qPCR may also be performed on AA-aRNA leftover from microarray experiments [Ambion Tips from the Bench, Using Excess Labeled aRNA for Microarray Validation, TechNotes Volume 14(1)]. This is particularly valuable when limited amount of RNA is available for validation - in case of research protocols using the PAXgene™ system for instance. However, care should be taken when designing such validation experiments. Here, we report our experience with microarray validation by qPCR on AA-aRNA and we present an optimized protocol that improves the reliability of this validation.

## Results and Discussion

### An alternative to total RNA to perform microarrays validation by qPCR

We took profit of an ongoing protocol of our lab which aimed at identifying new prognostic biomarkers of renal graft rejection. Hypothesizing that inflammation in the organ donnor conditions the success of transplantation [16], we analyzed the transcriptome of whole blood cells of brain-dead organ donors by microarrays. The hypothesis beyond this protocol was that graft rejection by the receiver may be predicted by the transcriptomic analysis of blood cells from the donor. Total RNA of whole blood cells collected in PAXgene™ tubes from 22 brain-dead organ donors was extracted. Since a limited volume of blood (2.5 mL) was withdrawn in these tubes, only a low amount of RNA could be extracted. After taking out 1 μg of total RNA for microarrays, only 15 donors had enough remaining total RNA to perform validation experiments by qPCR. In an attempt to find an alternative material than total RNA to perform validation experiments, we tested whether qPCR could be performed on AA-aRNA leftover from microarray experiments.

### Amplification and amino allyl labeling of RNA inhibits qPCR

**Figure 1:**
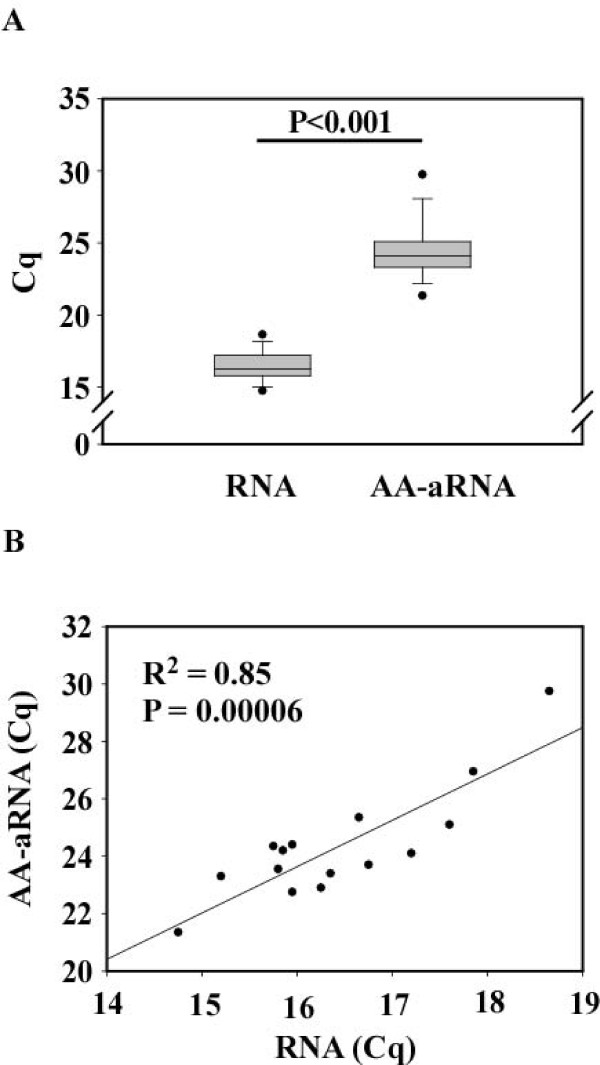
**Amplification and amino allyl labeling inhibit qPCR**. Total RNA extracted from whole blood cells of 15 healthy brain-dead organ donors was subjected to amplification by T7 polymerase and amino allyl labeling to generate AA-aRNA. 1 μg of un-amplified and un-labeled RNA and 100 ng AA-aRNA (amplified and amino allyl labeled RNA but not coupled with fluorescent dye) were reverse transcribed using SuperScript II, resulting cDNAs were diluted 10-fold and subjected to qPCR using primers specific for ACTB. (A) Cq obtained from the 15 samples are represented in a box plot. A statistically significant difference was detected between RNA and AA-aRNA (t = -25.23; P < 0.001; paired t-test). (B) Cq obtained with RNA and AA-aRNA for each of the 15 patients were strongly correlated (R^2 ^= 0.85; P = 0.00006; Pearson product moment correlation). Similar results were obtained in two independent experiments.

To compare the effectiveness of qPCR from total RNA and AA-aRNA, we used samples from the 15 donors for which we had both AA-aRNA and remaining total RNA. 1 μg RNA and 100 ng AA-aRNA were reverse transcribed and resulting cDNAs were diluted 10-fold before amplification by qPCR using primers specific for the ACTB gene. The rationale for using different amounts of RNA and AA-aRNA is that AA-aRNA is generated from only mRNA (a T7-Oligo(dT) primer being used during first strand DNA synthesis in the amplification protocol) whereas RNA includes all RNA species. The choice of 1 μg RNA and 100 ng AA-aRNA, as already used by others [17], was performed to represent the low proportion of mRNA in total RNA. We found that ACTB expression, as determined by Cq values, was lower when using AA-aRNA compared to RNA (Figure 1A). As a reminder, expression levels are inversely correlated to Cq value, which corresponds to the cycle of the qPCR for which a sufficient number of amplicons have accumulated to allow for a reliable quantification. Compiled from the 15 patients, Cq was 16.4 ± 1.0 (min-max 14.7-16.6) for RNA and 24.3 ± 2.0 (min-max 21.3-29.7) for AA-aRNA. This difference was highly significant (t = -25.2; P < 0.001). As shown in Figure 1B, there was a strong linear correlation between the Cq values obtained from RNA and AA-aRNA for each patient (R^2 ^= 0.85; P = 0.00006). These results attest that amplification and amino allyl labeling decrease the yield of qPCR without affecting its fidelity, most probably due to steric inderance engendered by incorporation of amino allyl arms. Therefore, the use of AA-aRNA to validate microarray results by qPCR is an attractive opportunity that requires optimization.

### Amplification and amino allyl labeling decrease qPCR sensitivity and efficiency

**Figure 2:**
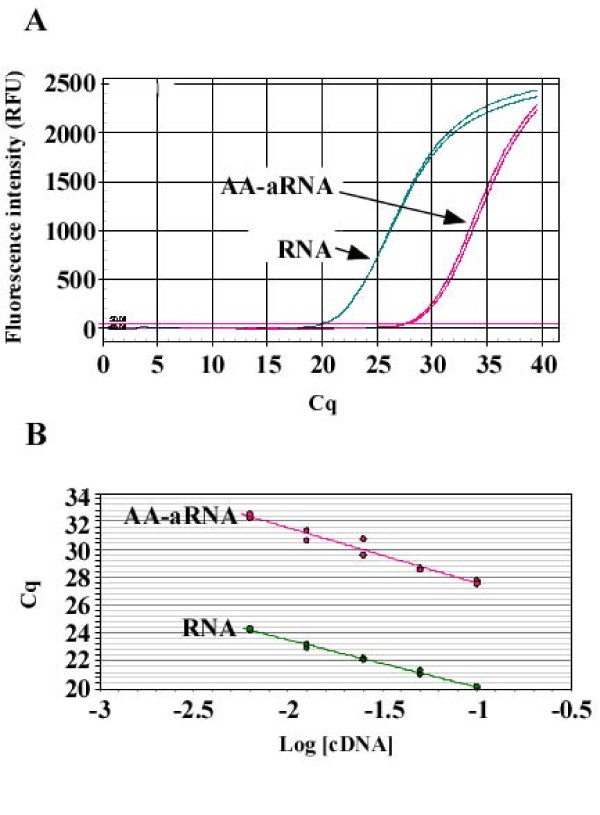
**Amplification and amino allyl labeling decrease qPCR efficiency and sensitivity**. One μg RNA and 100 ng AA-aRNA from human universal reference were reverse transcribed with SuperScript II. (A) Ten-fold dilutions of resulting cDNAs were subjected to qPCR using primer pairs specific for VEGFB. The amplification chart shows higher Cq values for AA-aRNA compared with RNA. (B) cDNAs obtained from reverse transcription of RNA and AA-aRNA were serially diluted and qPCR was performed to evaluate qPCR efficiency using VEGFB-specific primers. qPCR efficiency calculated from standard curves (E = [10^-1/slope^]-1) is down-regulated by amplification and amino allyl labeling.

Following experiments were performed with a universal reference RNA - the same as used for microarrays - because of its high quality and availability. This RNA is a commercially available mix of total RNA from 10 human cell lines. We first aimed to reproduce the inhibition of qPCR on AA-aRNA reported in Figure 1 with other genes than ACTB. Universal reference RNA was either amplified using T7 polymerase and coupled with amino allyl arms to generate AA-aRNA, or kept under its primary form (RNA). Both RNA and AA-aRNA were reverse transcribed with SuperScript II. Resulting cDNAs were diluted 10-fold and subjected to qPCR using primer pairs specific for 5 genes selected among our "genes of interest": vascular endothelial growth factor B (VEGFB), matrix metalloproteinase 9 (MMP9), transferrin receptor (TFRC), hepcidin antimicrobial peptide (HAMP), and glyceraldehyde-3-phosphate dehydrogenase (GAPDH). For instance, the rational for choosing VEGFB, a member of the family of angiogenic factors, came from the concept that angiogenesis plays a role in renal graft rejection [18]. Figure 2A displays VEGFB amplification chart for the two types of RNA. While this chart attested for the reliability of the amplification, we observed that Cq values increased from 20.1 for RNA to 27.7 for AA-aRNA (Figure 2A and Table 1). Similar results were obtained with the other genes (Table 1). Compiled from the 5 genes, Cq values were in average 5 cycles higher for AA-aRNA compared with RNA (P = 0.02). Interestingly, MMP9, which was readily detected when RNA was used as input (Cq = 27.3), could not be detected with AA-aRNA (Table 1). Then the two types of cDNAs were serially diluted before amplification by qPCR to evaluate qPCR efficiency. Analysis of VEGFB qPCR revealed that qPCR efficiency decreased from 97.3% for RNA to 79.1% for AA-aRNA (Figure 2B). Correlation coefficients of standard curves were typically > 0.98. These data show that amplification by T7 polymerase and incorporation of amino allyl arms inhibit both PCR sensitivity and efficiency. This inhibition may prevent the accurate quantification of low-abundant genes such as MMP9.

**Table 1:** Cq values obtained by qPCR using RNA and AA-aRNA

Gene	RNA	AA-aRNA
**VEGFB**	20.1	27.7
**MMP9**	27.3	ND
**TFRC**	18.4	24.6
**HAMP**	23.3	23.6
**GAPDH**	14.5	16.5
**Mean ± SD**	20.7 ± 4.9	25.6 ± 7.0 *

qPCR was performed on universal reference RNA and AA-aRNA using primer pairs specific for the indicated genes. ND: not detectable. * P = 0.02 vs RNA (one way repeated measures ANOVA).

### Optimized protocol for reverse transcription (RT)

**Figure 3:**
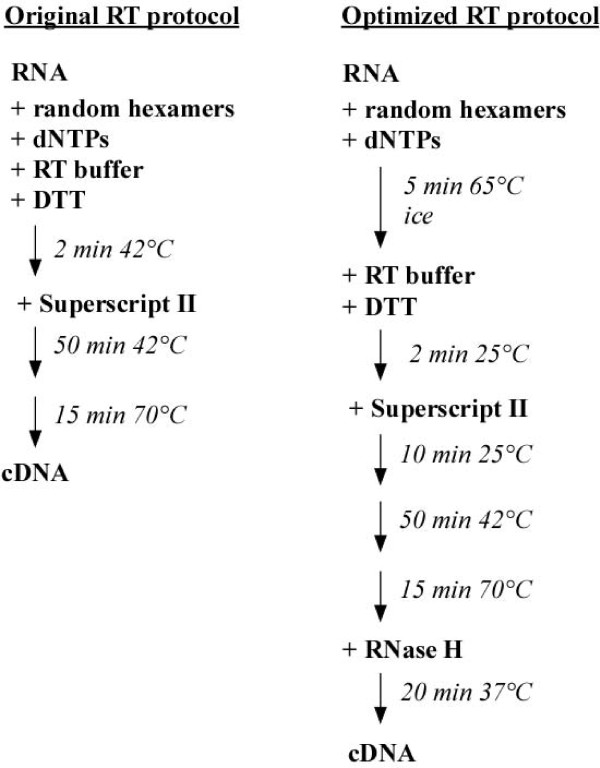
**Scheme depicting RT protocol optimizations**.

In an attempt to circumvent the loss of sensitivity of qPCR performed with AA-aRNA, we modified the RT protocol (Figure 3). We introduced a denaturation step (5 min at 65°C) followed by a rapid cool-down on ice and a 2-min incubation at 25°C to improve random hexamers annealing. After addition of reverse transcriptase, a transcription initiation step of 10 min at 25°C was performed to improve RT. This step is especially important when the RT is primed by random primers. Upon RT completion (50 min at 42°C) and enzyme denaturation (15 min at 70°C), samples were treated with RNase H for 20 min at 37°C to digest remaining RNA.

### Protocol optimization improves RT yield

**Figure 4:**
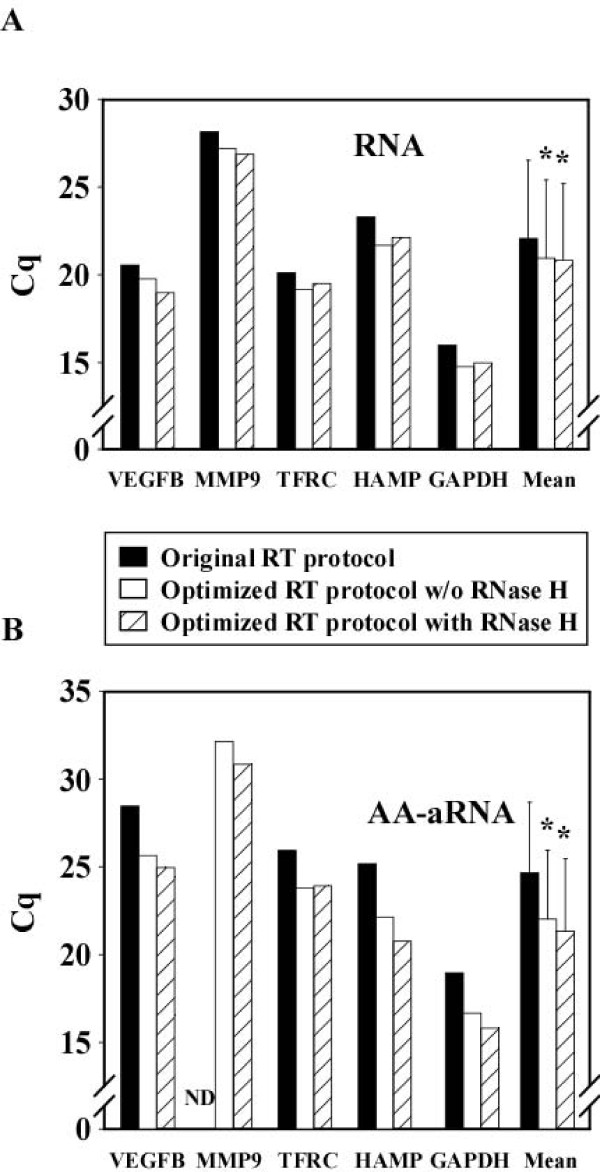
**Protocol optimization improves RT yield**. One μg RNA (A) and 100 ng AA-aRNA (B) obtained from universal reference RNA were used as inputs to either the original RT protocol (black bars), the optimized RT protocol without RNase H treatment (white bars), or the modified RT protocol with RNase H treatment (hatched bars). Resulting cDNAs were subjected to qPCR using primer pairs specific for VEGFB, MMP9, TFRC, HAMP and GAPDH. Threshold Cq values for each gene and the mean ± SD of the 5 genes are indicated. RT protocol optimization decreased Cq values and RNase H did not induce a further decrease. * P < 0.001 vs original RT protocol (one way repeated measures ANOVA). ND: not detectable.

To test RT protocol optimization, we used the universal reference RNA either under its native form (RNA) or after amplification and amino allyl incorporation (AA-aRNA). These 2 RNAs were reverse transcribed either with the original RT protocol, the optimized RT protocol without RNase H treatment, or the optimized RT protocol with RNase H treatment. This dichotomy allowed us to evaluate the effect of RNase H *per se*. qPCR was performed on resulting cDNAs using primer pairs recognizing the 5 genes VEGFB, MMP9, TFRC, HAMP and GAPDH. When RNA was used as starting material, the optimized RT protocol decreased the mean Cq value for the 5 genes by 1.2 cycle (from 22.0 ± 4.5 to 20.8 ± 4.3; P < 0.001) and RNase H treatment did not induce a further decrease of Cq value (Figure 4A). With AA-aRNA, the decrease of Cq value induced by RT protocol optimization was stronger: 2.6 cycles were gained (from 24. 8 ± 4.1 to 21.4 ± 4.2; P < 0.001). Again, RNase H treatment did not further decrease Cq values (Figure 4B). Interestingly, whereas MMP9 was below the detection threshold of qPCR assay with AA-aRNA and the original RT protocol (Cq > 35 cyles), protocol optimization was able to make it detectable (Figure 4B). The same experiment was repeated using RNA extracted from blood cells of one brain-dead organ donor and gave similar results: RT protocol modifications induced a gain of 1 cycle and 3.3 cycles when RNA and AA-aRNA were used as inputs, respectively (data not shown). These results show that optimization of the RT protocol improves the quantity of cDNA available for qPCR when working either with RNA or AA-aRNA, with a stronger effect with AA-aRNA.

### RNase treatment improves qPCR efficiency

**Figure 5:**
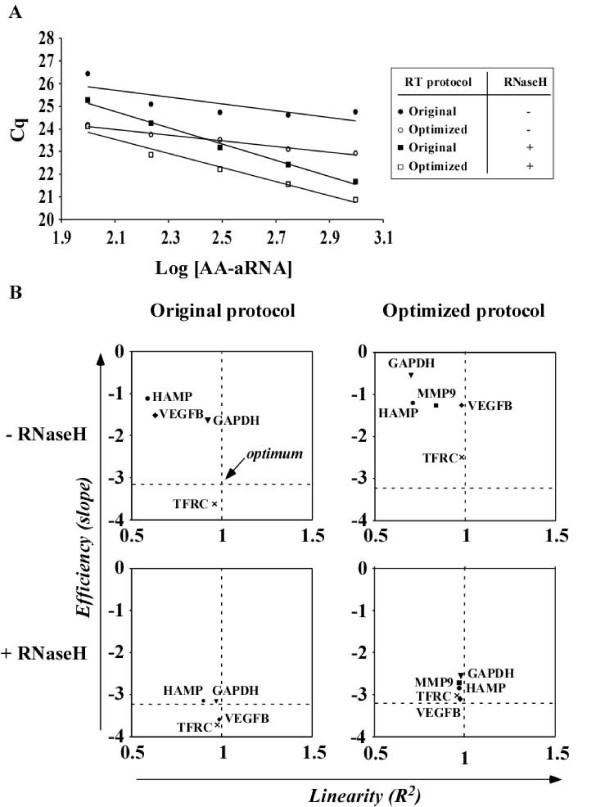
**RNaseH improves qPCR efficiency**. Different quantities of AA-aRNA from universal reference RNA (100-1000 ng) were used as inputs into either the original RT protocol or the optimized RT protocol, with or without RNase H treatment. Resulting cDNAs were diluted 10-fold and subjected to qPCR using primer pairs specific for VEGFB, MMP9, TFRC, HAMP and GAPDH. Cq values were plotted against the Log of the concentration of the AA-aRNA used for RT and linear regression was applied. qPCR efficiency (E) was calculated by the slope of the regression line. A slope of -3.2 indicates optimal efficiency. qPCR linearity (R^2^) corresponds to the correlation coefficient of the regression line. A coefficient R^2 ^of 1 indicates optimal linearity. (A) Representative experiment using VEGFB primers. (B) Plots representing qPCR efficiency as a function of linearity for the 5 genes tested. *Optimum *conditions are indicated at the intersection of dotted lines corresponding to E = -3.2 and R^2 ^= 1. E was improved by RNase H treatment.

We next investigated whether the optimized protocol and RNaseH treatment alter RT efficiency and linearity. It was indeed reported that RNase H may allow for amplifying certain genes which may not be accessible to PCR [19]. For this purpose, different quantities of AA-aRNA (100-1000 ng) were reverse transcribed using either the original or the optimized RT protocol, with or without RNase H treatment. Resulting cDNAs were diluted 10-fold and subjected to qPCR to determine the efficiency (E) and the linearity (R^2^) of the qPCR. Figure 5A displays the results of the qPCR for VEGFB and Figure 5B illustrates E and R^2 ^values obtained for the 5 genes tested with the different experimental protocols. Intersection of dotted lines in graphs of Figure 5B indicates the optimal values for E (-3.2) and R^2 ^(1). A gathering of genes near this *optimum *reveals an improvement of qPCR parameters. Table 2 gathers E and R^2 ^values for the 5 genes, together with statistical analyses. As shown in Figure 5, addition of RNase H to the original protocol improved qPCR efficiency for the 5 genes tested. When E values from the 5 genes were averaged, a significant improvement of E following RNase H treatment was found: from -1.96 ± 1.10 to -3.43 ± 0.30 (P = 0.003) when using cDNA generated by the original protocol, and from -1.36 ± 0.70 to -2.88 ± 0.29 (P < 0.001) when using cDNA generated by the optimized RT protocol (Table 2). E was not significantly improved by the optimized protocol if RNase H treatment was not performed (Table 2). Overall, RT protocol optimization (with RNase H) improved qPCR efficiency from -1.96 ± 1.10 to -2.88 ± 0.29 (P = 0.02). Linearity of the qPCR was not statistically significantly affected by the optimized protocol or RNase H (Table 2). However, protocol optimization and RNase H treatment clearly improved R^2 ^for selected genes such as VEGFB and HAMP (Figure 5 and Table 2).

**Table 2:** Effect of protocol optimization and RNase H on qPCR efficiency and linearity

	VEGFB	MMP9	TFRC	HAMP	GAPDH	Mean ± SD	*P vs -RNase H*	*P vs original protocol*	*P vs original protocol/- RNase H*
**Efficiency (slope)**	*Original protocol*								

- RNase H	-1.50	NA	-3.59	-1.15	-1.59	-1.96 ± 1.10			
+ RNase H	-3.74	NA	-3.63	-3.13	-3.24	-3.43 ± 0.30	0.003		

	*Optimized protocol*								
- RNase H	-1.13	-1.24	-2.53	-1.26	-0.65	-1.36 ± 0.70		0.13	
+ RNase H	-3.28	-2.83	-3.04	-2.71	-2.54	-2.88 ± 0.29	<0.001	0.16	0.02

**Linearity (R**^**2**^**)**	*Original protocol*								

- RNase H	0.66	NA	0.97	0.63	0.95	0.80 ± 0.18			
+ RNase H	0.99	NA	0.98	0.99	0.97	0.98 ± 0.01	0.16		

	*Optimized protocol*								

- RNase H	0.99	0.91	0.99	0.72	0.87	0.90 ± 0.11		0.38	
+ RNase H	0.98	0.97	0.97	0.99	0.98	0.98 ± 0.01	0.19	0.64	0.16

This table gathers the results obtained from experiments shown in Figure 5 and shows statistical analyses. *P *values were determined using one-way repeated measures ANOVA and paired t-test. NA: not applicable.

These data show that RNase H treatment improves qPCR efficiency. This result is consistent with previous data [20]. Considering that RNase H degrades RNA paired to cDNA after RT, improvement of qPCR parameters by RNase H suggests that RNA/cDNA duplexes may have prime amplification by Taq polymerase, inducing the synthesis of other PCR products than those targeted by the specific qPCR primers. However, the observation that RNase H does not modify Cq values (Figure 4) is consistent with a minor effect of RNA/cDNA duplexes on qPCR outcome. Also, fusion curves obtained for each qPCR consistently showed a single peak, attesting for the specificity of the amplification (not shown). Together with the previous observation that RT protocol optimization improved RT yield and qPCR sensitivity, these results demonstrate that our optimized RT protocol with RNase H treatment provides an optimal cDNA from AA-aRNA to be used in qPCR experiments.

### Importance of the distance from 3' end in the design of qPCR primers

**Figure 6:**
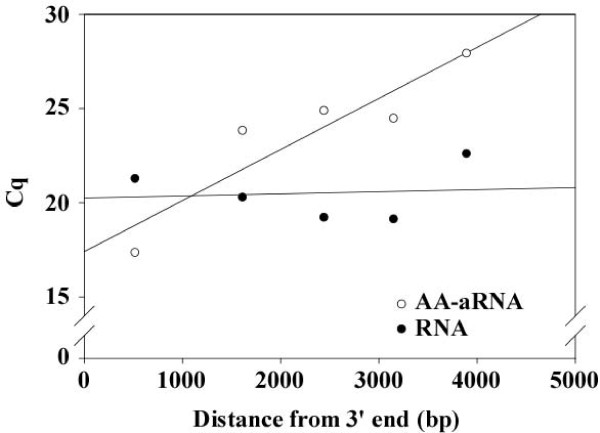
**Choice of primer location for qPCR from AA-aRNA**. One μg of universal reference RNA and 100 ng AA-aRNA were reverse transcribed using our optimized RT protocol. Resulting cDNAs were diluted 10-fold and subjected to qPCR using 5 pairs of primers located at different distances of the 3' end of the hif1a gene. Cq values were plotted against these distances. Linear regression lines illustrate the effect of increasing the distance from 3' end. Moving away primers from the 3' end increased Cq values when performing qPCR with cDNA generated from AA-aRNA.

Random hexamers used in traditional RT protocol bind anywhere in the RNA, allowing RT of all RNA independently of their size. In contrast, the RT of the amplification procedure that generates a AA-aRNA for microarrays is performed with T7 oligo(dT) primers that anneal only to the poly(A) tails of mRNA. This is an important limitation of this protocol since only mRNA below a certain length (typically around 1000 bp) may be correctly reverse transcribed, and therefore detectable by qPCR. To address this issue, we designed 5 pairs of primers located at different distances from the 3' end of the hif1a gene (Table 3). These primer pairs had similar ratings as determined by the Beacon software used for their design, ruling out the possibility that the differences observed could originate from the primers themselves. HIF1A was chosen for these experiments for the length of its coding sequence and the possibility to design 5' pairs of primers evenly distributed on this sequence. cDNAs generated from 1 μg RNA and 100 ng AA-aRNA from universal reference were diluted 10-fold and subjected to qPCR using HIF1A primers. As expected, a similar Cq value was obtained with the 5 pairs of primers when using RNA for qPCR, attesting that primer position does not affect Cq values when RT is performed with random hexamers (Figure 6). In contrast, moving away primers from the 3' end highly increased Cq values when using cDNA generated from AA-aRNA (Figure 6). These results highlight the importance of designing primer pairs close to the 3' end of the gene when working with AA-aRNA. We suggest designing qPCR primers closer than 1000 bp away from the 3' end of the target gene when performing qPCR from AA-aRNA.

**Table 3:** List of primers used in this study

Gene	cDNA length (bp)	Genbank accession number	Forward primer	Reverse primer	Distance from 3' end (bp)
ANXA1	1399	NM_000700	GGAACGCTTTGCTTTCTCTTG	TTCTGGTGGTAAGGATGGTATTG	795
ACTB	1852	NM_001101	AGAAAATCTGGCACCACACC	GGGGTGTTGAAGGTCTCAAA	1520
ELANE	938	NM_001972	CGGGCTAATCCACGGAATTG	TTGTCCTCGGAGCGTTGG	273
GAPDH	1310	NM_002046	CAGCCTCAAGATCATCAGCA	TGTGGTCATGAGTCCTTCCA	782
HAMP	430	NM_021175	AGTGGCTCTGTTTTCC	GAAGTGGGTGTCTCG	292
HIF1A	4082	NM_001530	AGAAGGTATGTGGCATTTATTTGG	CAGGGTAGGCAGAACATTTAGG	516
			CGTGTTATCTGTCGCTTTGAGTC	TTTCGCTTTCTCTGAGCATTCTG	1611
			TTGGCAGCAACGACACAG	GCAGGGTCAGCACTACTTC	2440
			AGCCGAGGAAGAACTATGAAC	ACTGAGGTTGGTTACTGTTGG	3149
			CCTGACAAGCCACCTGAG	TCGTGAGACTAGAGAGAAGC	3892
LY96	619	NM_015364	TGCCGAGGATCTGATGAC	ATTAGGTTGGTGTAGGATGAC	245
MMP9	2387	NM_004994	AACTACGACACCGACGAC	CAGGCGGAGTAGGATTGG	1587
STAT3	4900	NM_003150	GCTGGCTGACTGGAAGAG	AGTTGAGATTCTGCTAATGACG	3943
TFRC	5241	NM_003234	ATTGAACCTGGACTATGAGAG	GGAAGTAGCACGGAAGAAG	3140
TNF-α-1	1669	NM_000594	AGTGACAAGCCTGTAGCC	GGACCTGGGAGTAGATGAG	1243
TNF-α-2	1669	NM_000594	AAACAATGCTGATTTGGTGAC	GGCGATTACAGACACAACTCC	119
VEGFB	1172	NM_003377	CTGTGGTGGCTGCTG	ACTGGCTGTGTTCTTCC	901

Primers were designed with the Beacon Designer software. All PCR products were sequenced to confirm specificity.

### Validation of microarray results by qPCR using AA-aRNA

**Figure 7:**
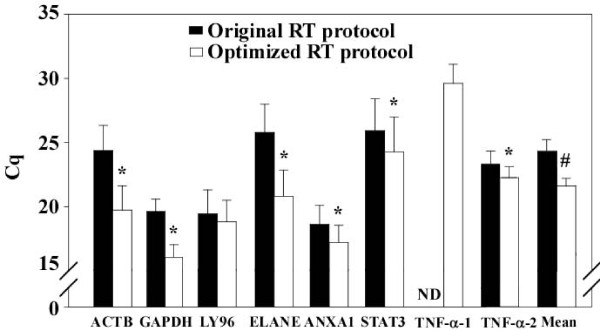
**RT protocol optimization decreases qPCR Cq values**. Total RNA extracted from blood cells of the same 15 brain-dead organ donors used in Figure 1 was amplified and amino allyl labeled to generate AA-aRNA. 100 ng of each of these AA-aRNA were then reverse transcribed to cDNA using either the original or the optimized RT protocol, and qPCR was applied to assess expression levels of ACTB, GAPDH, LY96, ELANE, ANXA1, STAT3 and TNF-α. Results are represented as Cq values for each gene (mean ± SD of the 15 donors) and as the mean ± SD of the 7 genes. The optimized RT protocol decreased Cq values. ND: not detectable. * P < 0.001 and # P = 0.004 vs original RT protocol (paired t-test).

As stated before, the ultimate goal of performing qPCR on AA-aRNA is to validate microarray results. We therefore tested whether the implementations described in this paper improved this validation. For this purpose, we used AA-aRNA obtained from blood cells of the same 15 brain-dead organ donors described earlier. On one hand, these 15 AA-aRNA were analyzed by genome-wide microarrays. Among the 25,000 genes represented on the microarrays, 5 genes were selected based on their significant association with renal graft rejection (analyses not shown here): the cytokine Tumor Necrosis Factor-α (TNF-α), the accessory protein LY96, the neutrophil elastase ELANE, the adhesion molecule annexin A1 (ANXA1), and the transcription factor Signal Transducer and Activator of Transcription 3 (STAT3). Expression levels obtained by microarrays were calculated as the log ratio sample vs universal reference RNA. On the other hand, expression levels of these genes were assessed by qPCR starting with the same 15 AA-aRNA that were used for microarrays. 100 ng of each AA-aRNA was reverse transcribed either with the original RT protocol or with the optimized RT protocol. The resulting two types of cDNA were subjected to qPCR using primers specific for ACTB, GAPDH, LY96, ELANE, ANXA1, STAT3 and TNF-α. Two pairs of primers were used for TNF-α, the first one (TNF-α-1) located 1243 bp away from the 3' end of the tnf-α gene and the second one (TNF-α-2) located 119 bp away from the 3' end (Table 3). Figure 7 depicts Cq values obtained by qPCR: for all genes, Cq values were lower when using AA-aRNA reverse transcribed with the optimized RT protocol compared with the original RT protocol. However, this effect failed to reach statistical significance for LY96. On average, a gain of 2.7 cycles was afforded by RT optimization (P = 0.004, Figure 7). This gain appeared to be independent of the primers position on the gene since some primers are located close to the 3'end (GAPDH, LY96, ELANE, ANXA1, TNF-α-2,) and others are located more distantly (ACTB, STAT3, TNF-α-1). However, the primer position was important in the case of TNF-α since the use of primers distant from the 3' end (TNF-α-1) prevented the detection of TNF-α in cDNA generated with the original RT protocol whereas primers located closely to the 3' end (TNF-α-2) allowed for TNF-α detection (Figure 7). This result illustrate that designing qPCR primers close to the 3' end is critical for specific target genes. In our search to identify new prognostic markers of graft rejection, the possibility to validate microarray data for TNF-α was important since TNF-α is a main pro-inflammatory cytokine and inflammation influences graft rejection [16]. Additionally, quantification of low-abundant genes is critical for biomarker studies since biomarkers are often low-abundant proteins encoded by weakly expressed mRNAs [21]. GAPDH was introduced in these analyses to avoid a possible inhibitory effect due to the location of ACTB primers (1520 bp away from the 3' end) and was chosen for normalization in subsequent experiments.

**Figure 8:**
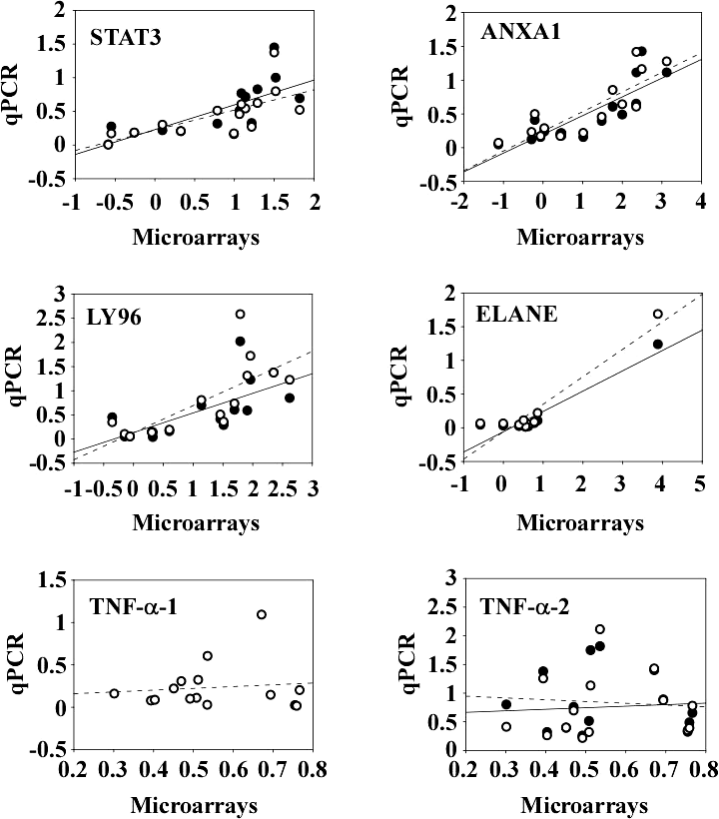
**Correlation between microarrays and qPCR**. Gene expression profiles of blood cells from 15 brain-dead organ donors were analyzed by microarrays covering 25,000-genes. Among these, the following were considered in these experiments: TNF-α, LY96, ELANE, ANXA1 and STAT3. Expression levels of these 5 genes obtained by microarrays were calculated as the log ratio sample vs universal reference RNA. 100 ng of AA-aRNA remaining from microarray experiments were reverse transcribed using either the original or the optimized RT protocol and assessed for expression levels of these genes by qPCR. GAPDH was used as reference gene for normalization. For each gene, expression data from microarrays (log ratio gene/reference) were plotted against expression data from qPCR calculated with the formula (2^Cq gene - Cq GAPDH^). Correlation between microarrays and qPCR, either using the original (●) or the optimized (○) RT protocol, were calculated by Pearson product moment correlation and are summarized in Table 4. Linear regression lines are displayed for correlations between microarrays and qPCR data obtained with the original (full lines) or the optimized (dotted lines) RT protocol. RT protocol optimization did not modify the correlation between microarrays and qPCR.

We next determined whether the optimized RT protocol affected the correlation between microarray results and qPCR data. For this purpose, expression levels obtained by qPCR were normalized to GAPDH and plotted against expression values obtained by microarrays (Figure 8). Linear regression was applied and correlation coefficients R^2 ^and P values as determined by the Pearson product moment correlation are gathered in Table 4. RT protocol optimization did not alter the level of correlation between microarrays and qPCR (Figure 8 and Table 4). Correlation coefficients were typically above 68% and flanked with P values below 0.01, attesting for a significant correlation between microarrays and qPCR data (Table 4). The strongest correlation was observed for ELANE (R^2 ^= 0.94) but this was due to one outlier. Interestingly, the observation that both qPCR and microarrays reported a high level of ELANE expression for this outlier argues for the effectiveness of qPCR to validate microarray data. This expression was also particularly elevated when qPCR was performed from un-amplified and un-labelled RNA (not shown). With respect to TNF-α, we failed to correlate qPCR data with microarrays, even with the optimized RT protocol and qPCR primers chosen close to the 3' end of the gene (Figure 8 and Table 4). This cannot be explained by a low level of expression of TNF-α since Cq values obtained by qPCR with TNF-α-2 primers were around 24 cycles (Figure 7). Two transcripts can be generated from the tnf-α gene, a large transcript with 4 exons and a smaller transcript having only 2 exons. One could have explained the absence of correlation between qPCR data and microarrays if the qPCR primers would have detected a different transcript than the oligonucleotide probe of the microarray. However, both TNF-α-1 and TNF-α-2 primers, as well as the microarray probe, recognize the large transcript. This result illustrates the difficulty to validate microarrays data by qPCR encountered for some genes, which may consequently be considered as false positives. Of note, TNF-α expression values obtained by microarrays did not correlate with qPCR performed on un-amplified and un-labelled RNA (not shown). Overall, the correlation between microarrays and qPCR on AA-aRNA obtained in our study is comparable to the correlations obtained with RNA ([22,23] and unpublished data). Therefore, qPCR data obtained from AA-aRNA are consistent with microarrays and our optimized RT protocol did not affect this correlation.

**Table 4:** Correlation between microarrays and qPCR

		STAT3	ANXA1	ELANE	LY96	TNF-α-1	TNF-α-2
**Microarrays vs original RT protocol**	R^2^	0.72	0.84	0.94	0.68	NA	-0.09
	*P*	0.002	0.0001	0.0002	0.005	NA	0.77
**Microarrays vs optimized RT protocol**	R^2^	0.69	0.84	0.94	0.73	0.11	0.07
	*P*	0.005	0.0001	0.0001	0.002	0.70	0.80

This table gathers correlation coefficients (R^2^) and *P *values estimated by Pearson product moment correlation from experiments shown in Figure 8. See legend to this figure for details. NA: not applicable.

## Conclusions

We have implemented an optimized protocol for the validation of microarrays data by qPCR. This protocol allows using AA-aRNA leftover from microarray experiments when limited amount of RNA is available. It can aid in the quantification of low-abundant genes and provides a significant level of correlation between microarrays and qPCR. In addition, this protocol provides high-quality material that can be used to validate expression of relevant genes that may be highlighted by other approaches than microarrays. Such approaches, some of them being increasingly used in the field of biomarker or therapeutic targets discovery, include bioinformatic analysis of functional networks or signaling pathways [24,25].

## Methods

### RNA samples

Two types of RNA were used in this study. First, RNA was extracted from whole blood cells of 22 brain-dead organ donors. Median age was 50 (36-64), 14 donors were males, and the initial events that led to brain death were cerebrovascular accident (n = 10), brain trauma (n = 6), acute onset of brain hypoxia (n = 2), suicide (n = 2) and trauma (n = 2). According to the French legislation, studies on brain-dead patients do not require informed consent. The French "Agence de la Biomédecine" approved the protocol and blood was withdrawn after signature of next of kin for all scientific studies of the brain-dead patient. Diagnosis of brain death was established according to the criteria of the French "Agence de la Biomédecine" [26]. Arterial blood (2.5 ml) was withdrawn from the arterial catheter in PAXgene™ blood RNA tubes (PreAnalytix^®^, BD Europe, Erembodegem, Belgium), in the operation room, just before organ harvesting. PAXgene™ tubes were stored at -20°C before RNA extraction. Total blood RNA was isolated using the PAXgene™ Blood RNA kit (Qiagen, Courtaboeuf, France) according to the manufacturer's instructions. RNA quantity was assessed with a Nanodrop (Thermo Scientific, Wilmington, USA) and quality was evaluated using the Agilent 2100 Bioanalyzer (Agilent Technologies, Palo Alto, CA). All RNAs used in the present study were of high quality and un-degraded (OD_260_/OD_280 _> 1.9 and OD_260_/OD_230 _> 1.7, RNA integrity number (RIN) > 8). Second, we used the universal human reference RNA comprising total RNA from 10 human cell lines (Stratagene Europe, Amsterdam, The Netherlands). All nucleic acid samples were stored at -80°C until use.

### RNA amplification and amino allyl labeling

Messenger RNAs were amplified using the Amino Allyl MessageAmp^® ^kit (Ambion, Cambridgeshire, United Kingdom) according to the manufacturer's protocol, starting with one μg of total RNA. This protocol is based on the Eberwine RNA amplification procedure [9]. Briefly, the generation of multiple antisense RNA copies of each mRNA is obtained by first strand cDNA synthesis with an oligo(dT) primer tagged with a T7 promoter. After second strand synthesis, an in vitro transcription reaction is performed with T7 RNA polymerase in presence of 5-(3-aminoallyl)-UTP (AA-UTP). This produces amplified amino allyl RNA (AA-aRNA) that can be coupled with fluorescent dyes for microarray experiments.

### Reverse transcription of RNA and AA-aRNA

The same RT protocol was applied to RNA (un-amplified and un-labeled RNA) and AA-aRNA (amplified and amino allyl labeled RNA but not coupled with fluorescent dye). 1 μg of RNA and 100 ng of AA-aRNA were reverse transcribed into cDNA using the SuperScript II reverse transcriptase (Invitrogen, Merelbeke, Belgium) with the following protocol: RNA or AA-aRNA was mixed with the 5× RT buffer, random hexamers, dNTPs and DTT in a total volume of 19 μl. Samples were then heated to 42°C for 2 min, and 1 μL of SuperScript II was added to a total volume of 20 μl. Final concentrations were: 50 mM Tris-HCl, 75 mM KCl, 3 mM MgCl_2_, 0.5 mM dNTPs, 10 mM DTT, 200 U of SuperScript II, 180 ng of random hexamers (Invitrogen). RT was allowed for 50 min at 42°C and was followed by enzyme inactivation at 70°C for 15 min. The absence of contaminating DNA was checked using no RT assays.

Optimized RT protocol. RNA or AA-aRNA were mixed with 180 ng random hexamers and dNTPs to a total volume of 13 μL, heated to 65°C for 5 min and rapidly chilled on ice for 5 min. 4 μl of 5× RT buffer and 2 μl DTT were then added and samples were incubated for 2 min at 25°C. Then 1 μL of Superscript II was added and samples were pre-incubated for 10 min at 25°C before RT for 50 min at 42°C. RT was stopped by heating to 70°C for 15 min. Finally, 1 μL (2 U) of RNase H (Ambion) was added, and incubation was continued for 20 min at 37°C. Reagent concentrations were the same as above.

### Quantitative real-time PCR (qPCR)

cDNAs obtained from RT of RNA or AA-aRNA were diluted 10-fold and 4 μL were mixed with 16 μL of SYBR^®^Green Master Mix (Biorad, Nazareth, Belgium) containing 300 nM of each primer (final volume 20 μL). Amplification was carried out in the IQ5 thermal cycler (BioRad) under the following conditions: heating for 3 minutes at 95°C, 40 cycles of denaturation for 30 seconds at 95°C, followed by an annealing/extension for 1 min. A negative control without cDNA template was run in every assay and measures were performed in duplicates. Primers were designed with the Beacon Designer Pro 7.0 software (Premier Biosoft) and their characteristics are indicated in Table 3. Primers specificity was assessed using the NCBI BLAST tool http://www.ncbi.nlm.nih.gov/BLAST/Blast.cgi. HPLC-purified primers were obtained from TIB MOLBIOL (Berlin, Germany). Expression levels were calculated using the freely available GENEX Expression Macro (Biorad) which takes into account primer efficiency. Melting curves were analyzed and amplicons were sequenced to confirm the specificity of the reaction. See 'additional file 1' for MIQE checklist.

### Microarrays

Transcriptomic profiles of whole blood cells from 22 brain-dead organ donors were obtained using oligonucleotide microarrays representing 25,000 genes [27]. Total RNA extracted from whole blood cells was used in combination with reference RNA (Universal Human Reference RNA) to provide an internal standard for comparisons of relative gene expression levels across arrays. Messenger RNAs were amplified using the Amino Allyl MessageAmp™ kit (Ambion^®^, Cambridgeshire, United Kingdom) according to the manufacturer's protocol, starting with one μg of total RNA. Five μg of each amino allyl aRNA were labeled with Cy3 or Cy5 (Amersham, Buckinghamshire, United Kingdom). Dye coupling to amino allyl aRNA was measured using the ND-1000 spectrophotometer. Dye coupling yield >5% was a prerequisite for further analysis. 750 ng of each amino allyl aRNA labeled Cy3 or Cy5 (reference RNA or donor RNA) were combined and hybridized on oligonucleotide microarrays representing 25,000 genes. Four microarrays per patient were hybridized and a dye-swap was performed (2 microarrays patient-Cy3/reference-Cy5 and 2 microarrays patient-Cy5/reference-Cy3). Hybridization steps were performed using the Agilent Technologies system. Briefly, RNA was fragmented with a fragmentation buffer before mixing with a hybridization buffer. Microarrays were blocked with 50 mM ethanolamine in 50 mM borate buffer pH = 9.0. Agilent's hybridization chambers and rotating oven were used for hybridization at 60°C for 17 h at 4 rpm. Microarrays were washed for 10 min in 6X SSC, 0.005% Triton X-102, for 5 min in 0.1X SSC, 0.005% Triton X-102, and were then dried by centrifugation before scanning using an Axon 4000B microarray scanner and the GenePix Pro 6^® ^software (Molecular Devices, Berks, UK). Self photomultiplicator gain adjustment and 0.1% saturated spots were allowed during scanning. Spot finding and raw data quantification of all four microarrays for each patient were performed in a batch analysis using the MAIA^® ^freeware. A Lowess non linear normalization step was performed with the Acuity^® ^software (Molecular Devices) to compensate for uneven Cy3-Cy5 distribution. The normalized log ratio Cy3/Cy5 was used in subsequent steps. A filtering step was then performed to remove genes that were not present in at least three microarrays out of four. The quality and reproducibility of each of the four microarrays per patient were evaluated using ANOVA, correlation coefficients and Self Organizing Maps drawn with the Acuity^® ^software. Data are stored in the Web based Microarray Data manager MEDIANTE and are available at the Gene Expression Omnibus database (http://www.ncbi.nlm.nih.gov/geo/) under the accession number GSE8723. Before statistical analysis, genes not present in at least 50% of the patients were filtered out. Supervised analysis was performed using the Significance Analysis of Microarrays (SAM) software which correlates gene expression with an external variable such as EF value. Two class unpaired t-test and 100 permutations were used. Gene missing values imputation was performed via a K Nearest Neighbour algorithm normalization using 10 neighbours.

### Statistical analysis

Results are presented as mean ± SD or as median (interquartile range) for description of demographic characteristics. Comparisons between two groups were performed with two-tailed t-test for Gaussian data and Mann-Whitney test for non Gaussian data. Comparisons between multiple groups were performed with one way ANOVA for Gaussian data and Kruskal-Wallis one way ANOVA on ranks for non Gaussian data. Paired data among multiple groups were compared with one way repeated measures ANOVA and all pairwise multiple comparison procedures (Holm-Sidak method). Correlation tests were performed using the Pearson product moment correlation method. Statistical significance tests were generated with the SigmaPlot v11.0 software and the SigmaStat software (for Windows version 3; SPPS Inc. Chicago, Illinois, USA). A *P *value < 0.05 was considered statistically significant.

## Authors' contributions

CJ and YD jointly designed the study and developed the optimized RT protocol. CJ carried out the experiments. YD drafted the manuscript. DL and PMM participated in the design of the study and clinical sample collection. DRW provided a critical review of the manuscript. All authors participated in the drafting of the manuscript. All authors read and approved the final manuscript.

## Supplementary Material

Additional file 1 **MIQE_checklist**. Minimum information for publication of quantitative real-time PCR experiments guidelines. Click here for file
